# Editorial: Hormones and person perception

**DOI:** 10.3389/fpsyg.2025.1637860

**Published:** 2025-06-18

**Authors:** Lisa L. M. Welling, Amanda C. Hahn, Iris J. Holzleitner

**Affiliations:** ^1^Department of Psychology, Oakland University, Rochester, MI, United States; ^2^Department of Psychology, Cal Poly Humboldt, Humboldt, CA, United States; ^3^School of Social Sciences, University of the West of England Bristol, Bristol, United Kingdom

**Keywords:** hormones, person perception, testosterone, cortisol, progesterone, estrogen

Recent research bridging behavioral endocrinology and social psychology has refined our understanding of how hormones influence person perception (see Welling and Shackelford, 2019). This Research Topic on *Hormones and Person Perception* brings together empirical studies, methodological insights, and reviews advancing understanding of how hormones can influence perceptions and expressions of traits like attractiveness, health, and social behaviors related to relationship dynamics. The nine accepted papers reflect diverse approaches. Three priorities emerge: hormonal effects involve complex interactions with individual differences and social cues, methodological rigor is vital, and research should integrate genetic, behavioral, and environmental factors to better capture biological foundations of social cognition.

## Context-dependent effects

Goetz et al. tested whether exogenous testosterone affects men's perceptions of a woman's sexual interest. The sexual misperception bias (SMB), where men overestimate women's sexual interest, has been interpreted through Error Management Theory (Haselton and Buss, 2000), which proposes that, for men, misperceiving sexual interest carried fewer reproductive costs ancestrally than did missing potential mating opportunities (Buss, 2001). In this placebo-controlled experiment, testosterone administration did not increase SMB, but did heighten sensitivity to affiliative cues, especially among men with average or higher self-perceived attractiveness. Testosterone-treated men who interpreted a woman's behavior as affiliative perceived greater sexual interest, an effect not observed among placebo-treated men. These results suggest testosterone functions as a social hormone shaping perceptual biases in specific contexts.

Donovan and Corpuz explored how testosterone, cortisol, and relationship satisfaction relate in first-time fathers during the postpartum period. Fathers with high testosterone and low cortisol reported higher relationship satisfaction, although this effect was small. The authors suggest that traits often associated with high testosterone, such as dominance and status-seeking (reviewed in Dekkers et al., 2019), may also influence relationship quality beyond mate acquisition. This study suggests complex hormonal influences on relationship dynamics and calls for more precise, multi-method, longitudinal research surrounding the transition to parenthood.

Using an eye-tracking paradigm, Garza and Byrd-Craven investigated women's visual attention to facial masculinity across the menstrual cycle. Contrary to the Ovulatory Shift Hypothesis (Gangestad and Thornhill, 1998), although women spent more time viewing masculine than feminine faces, particularly in a long-term mating context, no hormone measures predicted visual attention to or rated preference for masculine faces across the menstrual cycle. There was partial evidence linking women's hormone levels to visual attention. Higher estradiol to progesterone ratios were associated with shorter first fixations, whereas lower progesterone predicted greater visual attention to male faces in a short-term mating context. Also, higher estradiol levels were related to more overall visual fixations. Findings emphasize that hormonal influences on social cognition may be subtler or more context-dependent than assumed.

Similarly, Lobmaier et al. recorded women during high- and low-fertility phases while reading and reproducing spoken sentences from male and female speakers rated as either attractive or unattractive. Vocal parameters varied by cycle phase, in response to the stimulus speaker's vocal attractiveness and sex, and when reproducing spoken sentences compared to reading written sentences. Women also used breathier, higher-frequency voices when responding to attractive voices, consistent with social mimicry research (Chartrand and Lakin, 2013). In contrast, Friedrich et al. found no cycle phase or hormonal effects on voice-gender categorization, though participants responded faster to feminine voices (see also Lattner et al., 2005). These null results align with emerging reports of weak or inconsistent cycle effects on female social cognition (e.g., Garza and Byrd-Craven, 2019; Jones et al., 2018). Inconsistent findings highlight the need for precise hormone measurement, methodological rigor, and considering other potential sources of variation across studies.

## Methodological rigor

Hampson et al. assessed depression in women during the active hormone phase and the hormone-free “washout” week of their contraceptive cycles. The study combined explicit self-reports with implicit measures of depressed affect, finding that implicit measures yielded a pattern of increased depressive affect during active hormone intake, particularly among those who report higher average levels of depressive affect. In contrast, explicit self-reports indicated that participants perceived greater depressive affect when taking inactive pills containing no synthetic hormones. These findings demonstrate the importance of implicit measures for capturing mood effects not detected in self-report measures (e.g., DeCoster et al., 2006) and suggest OC-related mood effects may be most evident in those prone to depression.

Updating earlier work (Grimbos et al., 2010), Swift-Gallant et al. conducted a comprehensive meta-analysis examining second-to-fourth digit ratio (2D:4D), which is thought to be a marker of prenatal androgen exposure (see Swift-Gallant et al., 2020), and sexual orientation. The authors found that homosexual women tend to have lower (more male-typical) digit ratios than heterosexual women, whereas homosexual men exhibited higher (more female-typical) digit ratios than heterosexual men. No significant differences were found for bisexual individuals. These findings add to research on prenatal androgen exposure in sexual orientation development (Swift-Gallant et al., 2021) and suggest future work include multiple biological measures and nuanced sexual orientation categories.

## Expanding conceptual frameworks

Gurguis et al. proposed a quantitative genetic framework for studying the evolutionary dynamics of hormone-mediated traits, focusing on person perception and psychiatric conditions using estrogen as an example. The authors argue that person perception is part of broader hormone-regulated suites and should be studied within multivariate evolutionary models that account for genetic correlations among traits (McGlothlin and Ketterson, 2008). Quantitative genetics techniques could test hypotheses about selection on hormone-mediated traits and clarifying trade-offs, like estrogen's dual influence on reproductive fitness (Mittal et al., 2014) and disease risk (Chuffa et al., 2017). This framework offers a promising tool for connecting evolutionary biology, psychology, and psychiatry.

Arnocky and Davis investigated whether male facial attractiveness is related to health through shared hormonal and lifestyle factors, as suggested by Jones et al. (2021), rather than serving as a direct cue to immunocompetence. They measured immunoglobulin A, testosterone, cortisol, lifestyle behaviors, abdominal skinfold, and self-reported health alongside female-rated facial attractiveness. Abdominal skinfold and symptoms of poorer health predicted lower facial attractiveness, and mediated the relationships between exercise, stress, and facial attractiveness. Men with higher testosterone and lower cortisol tended to have more attractive faces, but this was not statistically significant. Results suggest facial attractiveness-immunocompetence associations may partly reflect shared lifestyle and hormonal factors, although inclusion of broader lifestyle measures is warranted.

## Conclusion

The above research offers valuable insights into how hormones shape person perception and introduce new priorities for future research. Future work should improve hormone measurement, broaden conceptual frameworks, and examine how individual differences moderate hormonal effects. Together, the published works in this Research Topic underscore the importance of considering hormones in person perception research and advance our understanding of the biological foundations of social cognition.

## References

[B1] Buss D. M. (2001). Cognitive biases and emotional wisdom in the evolution of conflict between the sexes. Curr. Dir. Psychol. Sci. 10, 219–223. 10.1111/1467-8721.00153

[B2] Chartrand T. L. Lakin J. L. (2013). The antecedents and consequences of human behavioral mimicry. Annu. Rev. Psychol. 64, 285–308. 10.1146/annurev-psych-113011-143754 23020640

[B3] Chuffa L. G. Lupi-Junior L. A. Costa A. B. Amorim J. P. Seiva F. R. (2017). The role of sex hormones and steroid receptors on female reproductive cancers. Steroids 118, 93–108. 10.1016/j.steroids.2016.12.011 28041951

[B4] DeCoster J. Banner M. J. Smith E. R. Semin G. R. (2006). On the inexplicability of the implicit: Differences in the information provided by implicit and explicit tests. Soc. Cogn. 24, 5–21. 10.1521/soco.2006.24.1.5

[B5] Dekkers T. J. van Rentergem J. A. A. Meijer B. Popma A. Wagemaker E. Huizenga H. M. (2019). A meta-analytical evaluation of the dual-hormone hypothesis: does CORT moderate the relationship between testosterone and status, dominance, risk taking, aggression, and psychopathy? Neurosci. Biobehav. Rev. 96, 250–271. 10.1016/j.neubiorev.2018.12.004 30529754

[B6] Gangestad S. W. Thornhill R. (1998). Menstrual cycle variation in women's preferences for the scent of symmetrical men. Proc. Royal Soc. London: Biol. Sci. 265, 927–933. 10.1098/rspb.1998.0380 9633114 PMC1689051

[B7] Garza R. Byrd-Craven J. (2019). Fertility status in visual processing of men's attractiveness. Evol. Psychol. Sci. 5, 328–342. 10.1007/s40806-019-00190-4

[B8] Grimbos T. Dawood K. Burriss R. P. Zucker K. J. Puts D. A. (2010). Sexual orientation and the second to fourth finger length ratio: a meta-analysis in men and women. Behav. Neurosci. 124:278. 10.1037/a0018764 20364887

[B9] Haselton M. G. Buss D. M. (2000). Error management theory: a new perspective on biases in cross sex mind reading. J. Pers. Soc. Psychol. 78:81. 10.1037/0022-3514.78.1.81 10653507

[B10] Jones B. C. Hahn A. C. Fisher C. I. Wang H. Kandrik M. Han C.. (2018). No compelling evidence that preferences for facial masculinity track changes in women's hormonal status. Psychol. Sci. 29, 996–1005. 10.1177/0956797618760197 29708849 PMC6099988

[B11] Jones B. C. Holzleitner I. J. Shiramizu V. (2021). Does facial attractiveness really signal immunocompetence? Trends in Cognit. Sci. 25, 1018–1020. 10.1016/j.tics.2021.09.003 34625347

[B12] Lattner S. Meyer M. E. Friederici A. D. (2005). Voice perception: Sex, pitch, and the right hemisphere. Hum. Brain Mapp. 24, 11–20. 10.1002/hbm.20065 15593269 PMC6871712

[B13] McGlothlin J. W. Ketterson E. D. (2008). Hormone-mediated suites as adaptations and evolutionary constraints. Philos. Trans. R. Soc. B, Biol. Sci. 363, 1611–1620. 10.1098/rstb.2007.0002 18048296 PMC2606720

[B14] Mittal S. Gupta P. Malhotra N. Singh N. (2014). Serum estradiol as a predictor of success of in vitro fertilization. J. Obstet. Gynecol. India 64, 124–129. 10.1007/s13224-013-0470-7 24757341 PMC3984655

[B15] Swift-Gallant A. Di Rita V. Major C. A. Breedlove C. J. Jordan C. L. Breedlove S. M. (2021). Differences in digit ratios between gay men who prefer receptive versus insertive sex roles indicate a role for prenatal androgen. Sci. Rep. 11:8102. 10.1038/s41598-021-87338-0 33854100 PMC8046970

[B16] Swift-Gallant A. Johnson B. A. Di Rita V. Breedlove S. M. (2020). Through a glass, darkly: Human digit ratios reflect prenatal androgens, imperfectly. Horm. Behav. 120:104686. 10.1016/j.yhbeh.2020.104686 32014464

[B17] Welling L. L. M. Shackelford T. K. (2019). The Oxford Handbook of Evolutionary Psychology and Behavioral Endocrinology. New York, NY: Oxford University Press.

